# Bridging the Gap: Soft Tissue Considerations in Dental Implants From Microscopic to Macroscopic Levels

**DOI:** 10.7759/cureus.64002

**Published:** 2024-07-07

**Authors:** Madhurima Sharma, Taniya Bhatia, Rohit Sharma, Rohit Nandan, Arpita Bagga, Harshita Kapoor

**Affiliations:** 1 Prosthodontics and Crown & Bridge, Teerthanker Mahaveer Dental College and Research Centre, Moradabad, IND; 2 Conservative Dentistry and Endodontics, Teerthanker Mahaveer Dental College and Research Centre, Moradabad, IND

**Keywords:** macroscopic factors, microscopic factors, gingival aesthetics, biocompatibility, dental implants, soft tissue integration

## Abstract

Successful dental implant therapy not only relies on osseointegration but also on the health and stability of the surrounding soft tissues. Soft tissue concerns are critical to the long-term success of dental implants, influencing both function and appearance. This review looks at soft tissue integration with dental implants from both microscopic and macroscopic viewpoints. It investigates the biological mechanisms, therapeutic management, and factors that influence soft tissue health around implants. By exploring these issues, the review hopes to provide a full understanding of the importance of soft tissue considerations in dental implantology.

## Introduction and background

Dental implants have become a common option for tooth replacement, providing dependable and long-term results. While the focus has typically been on obtaining strong osseointegration, the role of peri-implant soft tissues is receiving more attention [1,2]. Healthy soft tissue integration is critical for avoiding peri-implant illnesses, maintaining aesthetics and stability, and assuring overall implant success. Soft tissue integration at the microscopic level is a complex biological process that ensures the stability, function, and aesthetics of dental implants [3-5]. This study will look at soft tissue considerations in dental implantology from both microscopic and macroscopic views, providing insights into the elements that influence soft tissue health as well as techniques for achieving the best clinical outcomes.

## Review

Microscopic perspective

Cellular and Molecular Mechanisms

Soft tissue integration around dental implants involves a complex interplay of cellular and molecular processes.

Fibroblasts: These cells are essential for the development and maintenance of connective tissue around implants. They generate collagen and other extracellular matrix (ECM) components that aid in structural support [6,7].

Collagen production: Fibroblasts produce type I and III collagen, which make up the majority of the connective tissue around implants [7].

Extracellular matrix remodeling: These cells continuously reconstruct the ECM, maintaining tissue integrity and function over time [8].

Epithelial Cells

These cells act as a barrier to microbial infiltration and help to seal the implant-abutment contact.

Junctional epithelium: These cells form a seal around the implant by adhering to the titanium or zirconia surface with hemidesmosomes. This adhesion is critical for shielding the surrounding tissues from bacterial penetration [9,10].

Barrier function: The epithelial barrier is vital for preventing peri-implantitis and other infections.

Inflammatory Cells

During the first healing phase, macrophages, neutrophils, and lymphocytes contribute to the inflammatory response and impact soft tissue integration quality.

Macrophages: These cells have two roles, i.e., inflammatory response and tissue remodeling. They help to eliminate debris and organize the healing process by producing cytokines and growth factors [11,12].

Neutrophils and lymphocytes: These cells play a key role in the immediate immune response and infection control throughout the early phases of recovery [13].

Histological Features

Microscopic analysis reveals critical histological features of peri-implant soft tissues.

Junctional epithelium: The junctional epithelium (JE) that surrounds dental implants forms a seal, holding microbes and debris from accessing peri-implant tissues and supporting the immune response with immune cell content. The JE adheres to the implant surface via hemidesmosomes and the basal lamina and forms a protective shield against microbial invasion, which ensures implant stability. It also helps with tissue integration and healing, which promotes the implant's long-term success. By controlling the inflammatory response and maintaining a healthy epithelial barrier, the JE promotes gingival health and avoids peri-implantitis.

Attachment mechanism: Hemidesmosomes tie basal epithelial cells to the implant surface, producing a strong but flexible connection [14,15].

Barrier function: The JE serves as the initial line of defense, keeping microorganisms from penetrating the connective tissue and bone.

Connective Tissue Zone

This zone provides mechanical stability and support and is made up of collagen fibers, primarily type I and III, that are aligned parallel to the implant surface. This orientation differs from the perpendicular fiber arrangement found surrounding normal teeth.

Collagen fiber orientation: Parallel fibers add mechanical stability and help to distribute occlusal forces uniformly.

Vascularization: Adequate blood flow inside the connective tissue zone is critical for nutrient delivery and waste elimination, promoting tissue health and regeneration.

Blood Supply

Adequate vascularization is essential for nutrient delivery, waste removal, and healing. The microvasculature network around implants is crucial for maintaining healthy soft tissues [16].

Angiogenesis: The formation of new blood vessels is a key component of the healing process. Growth factors, such as vascular endothelial growth factor (VEGF), play a pivotal role in angiogenesis [17].

Nutrient delivery: An adequate blood supply ensures the delivery of essential nutrients and oxygen to the cells, facilitating tissue repair and maintenance.

Material Influence on Soft Tissue Response

Surface characteristics: The surface topography and chemistry of dental implants significantly influence soft tissue integration [18,19].

Smooth Versus Rough Surfaces

Smooth surfaces tend to accumulate less plaque, reducing the risk of peri-implantitis. Rough surfaces, on the other hand, enhance soft tissue attachment by increasing surface area and promoting fibroblast adhesion.

Surface Coating

Various surface coatings, such as hydroxyapatite or bioactive glass, can further improve tissue response by enhancing biocompatibility and promoting cell adhesion [20,21].

Implant Material

The choice of implant material affects the soft tissue response at the microscopic level.

Titanium: Titanium implants have a proven track record of successful soft tissue integration due to their biocompatibility and ability to form a stable oxide layer [22-24].

Zirconia: Zirconia implants, with their tooth-like color and excellent biocompatibility, promote healthy soft tissue attachment and reduce the risk of mucosal discoloration [22,23].

Biocompatibility and Soft Tissue Health

Biocompatibility is crucial for optimal soft tissue integration. Implant materials and surfaces must support cellular activities while causing no unwanted effects.

Cytocompatibility: The capacity of implant materials to facilitate cell adhesion, proliferation, and differentiation is critical for soft tissue integration [25,26].

Inflammatory reaction: Reducing the inflammatory response is critical for tissue health. Biocompatible materials lower the risk of chronic inflammation and peri-implant illness.

Table 1 shows the microscopic factors influencing the relationship between soft tissue and implant.

**Table 1:** Microscopic factors influencing the relation between soft tissue and implant. References [6-26].

Microscopic factor	Description	Relation to soft tissue integration
Fibroblasts	Cells that produce collagen and other extracellular matrix components.	Provide structural support, enhance tissue attachment, and contribute to the stability and maintenance of connective tissue.
Epithelial cells	Cells that form a barrier against microbial invasion and contribute to tissue attachment.	Form the junctional epithelium, attach to the implant surface via hemidesmosomes, and create a protective seal.
Macrophages	Immune cells involved in the inflammatory response and tissue remodeling.	Clear debris, release cytokines and growth factors, orchestrate healing, and influence tissue health around implants.
Neutrophils and lymphocytes	Immune cells involved in the immediate immune response.	Control infection during early healing stages and help in maintaining tissue integrity.
Junctional epithelium	Epithelial attachment to the implant surface, forming a seal.	Protects underlying tissues from bacterial infiltration and maintains the health of peri-implant tissues.
Connective tissue zone	Zone characterized by collagen fibers oriented parallel to the implant surface.	Provides mechanical stability, supports tissue attachment, and helps in the distribution of masticatory forces.
Blood supply	Microvascular network within peri-implant tissues.	Ensures nutrient delivery and waste removal, and supports healing and tissue maintenance.
Surface characteristics	Topography and chemistry of the implant surface (smooth vs. rough).	Influences plaque accumulation, fibroblast adhesion, and overall tissue response.
Surface coatings	Various coatings (e.g., hydroxyapatite and bioactive glass) applied to implant surfaces.	Enhance biocompatibility, promote cell adhesion, and improve soft tissue attachment.
Implant material	Types of materials used for implants (e.g., titanium and zirconia).	Affects biocompatibility, tissue response, and aesthetic outcomes; zirconia improves soft tissue aesthetics.
Cytocompatibility	The ability of implant materials to support cell functions.	Essential for cell adhesion, proliferation, and differentiation, contributing to successful soft tissue integration.
Inflammatory response	The immune response to implant placement and materials.	Minimizing chronic inflammation is crucial for maintaining tissue health and preventing peri-implant diseases.
Angiogenesis	Formation of new blood vessels around the implant.	Critical for healing, nutrient delivery, and maintaining healthy peri-implant tissues.
Collagen production	Synthesis of collagen by fibroblasts.	Key for forming the connective tissue matrix and providing structural integrity and support to soft tissues.
Extracellular matrix remodeling	Continuous remodeling of the extracellular matrix by fibroblasts.	Ensures the integrity and function of peri-implant tissues over time, adapting to mechanical and biological changes.

Macroscopic perspectives

Clinical Management of Soft Tissues

Surgical techniques: Effective surgical procedures are critical for maintaining and improving soft tissue integration around dental implants.

Minimally invasive surgery: Flapless surgery minimizes tissue stress and preserves soft tissue architecture. This approach involves inserting the implant through a tiny incision, reducing disruption of the peri-implant tissues and facilitating faster healing [27].

Lasers: Using lasers in dental implant surgery improves precision and control, resulting in targeted tissue removal with minimal influence on the surrounding areas. Lasers have antibacterial qualities that assist in lowering the risk of infection, and their ability to coagulate blood vessels reduces bleeding and gives a clearer surgical area. Due to less tissue damage, patients have faster healing and less postoperative pain. Furthermore, the minimally invasive nature of laser surgery leads to smaller incisions, less scarring, and a lower chance of problems, eventually boosting patient comfort and satisfaction.

Flap design: Proper flap design and handling are essential. The papilla preservation flap and the roll flap can preserve soft tissue volume and aesthetics [28].

Suturing techniques: These should also be enhanced, such as the adaptation of double-crossed sutures can be considered a suitable suturing technique in situations like surgical thickening of gingiva, implant second-stage surgery, gingival recession coverage, and soft tissue augmentation [29].

Soft tissue grafting: When soft tissue volume is low, grafting procedures might be used. Connective tissue grafts, free gingival grafts, and acellular dermal matrix grafts are frequently utilized to improve the thickness and quality of peri-implant [30].

Prosthetic Considerations

The design and placement of prosthetic components significantly impact soft tissue health and aesthetics.

Abutment selection: Abutment material and design impact soft tissue response. Custom abutments provide better adaptability to soft tissues than stock abutments. Furthermore, the use of zirconia abutments can improve cosmetic results by removing the danger of metal show-through [31].

A well-designed emergence profile promotes the natural contouring of soft tissues, resulting in a smooth transition from implant to restoration. Proper emergence profiles help to evenly distribute masticatory pressures and prevent soft tissue recession [31].

Proper prosthetic margin placement is crucial for maintaining soft tissue health. Subgingival margins should be avoided to limit the likelihood of plaque buildup and peri-implantitis. Supragingival or equigingival margins are preferred to provide good dental hygiene [32].

Maintenance and Monitoring

The long-term success of dental implants requires diligent maintenance and monitoring of peri-implant soft tissues.

Hygiene protocols: Proper oral hygiene is crucial for preventing peri-implant illnesses. Patients should be taught proper brushing and interdental cleaning practices. Professional cleaning should be done on a regular basis to eliminate plaque and calculus buildup [33].

Clinical assessments: Regular examinations of soft tissue health are important. Clinicians should keep an eye on probing depths, bleeding while probing, and any visible symptoms of inflammation. Early diagnosis of soft tissue problems enables timely intervention and therapy.

Aesthetic Outcomes

Achieving optimal aesthetics is a key goal in implant dentistry, particularly in the anterior region.

Gingival margin position: The position of the gingival margin relative to the implant affects the visual harmony of the smile. Careful consideration of the gingival margin during implant placement and restoration can enhance aesthetic outcomes.

Soft tissue contours: Symmetrical and natural-looking soft tissue contours around implants contribute to a pleasing appearance. Techniques such as papilla regeneration and the use of provisional restorations can help shape the soft tissues during the healing phase.

Factors Affecting Soft Tissue Health

Patient-related factors: Several patient-related factors influence soft tissue outcomes around dental implants. Systemic health conditions like diabetes and smoking might hinder soft tissue healing and integration. Managing these issues through medical intervention and patient education is critical for positive results. Genetic factors can impact soft tissue responsiveness and susceptibility to peri-implant disorders. Personalized treatment plans that take into account hereditary characteristics can improve soft tissue outcomes [34,35].

Implant-related factors: The design and placement techniques of dental implants are critical for optimal soft tissue integration.

Implant diameter and length: Selecting the appropriate implant diameter and length based on the clinical circumstances provides adequate support for soft tissues. Wide-diameter implants may give superior support in places with the thin soft tissue biotype [36,37].

Placement depth: Proper vertical placement of the implant is essential for maintaining biological width and avoiding soft tissue recession. Implants should be placed at a depth that provides adequate soft tissue covering and support [36,37].

Table 2 shows the macroscopic factors influencing the relation between soft tissue and implant.

**Table 2:** Macroscopic factors influencing the relation between soft tissue and implant. References [27-37].

Macroscopic factors	Description	Relation to soft tissue integration
Surgical techniques	Methods and approaches used during implant placement.	Minimize tissue trauma, preserve soft tissue architecture, promote faster healing, and reduce the risk of complications.
Minimally invasive surgery	Techniques such as flapless surgery reduce tissue disruption.	Preserve soft tissue volume, reduce healing time, and enhance tissue integration and aesthetics.
Flap design	Design and handling of surgical flaps during implant placement.	Maintain soft tissue volume and aesthetics, support proper healing and integration.
Soft tissue grafting	Procedures to enhance soft tissue volume and quality (e.g., connective tissue grafts).	Improve peri-implant mucosa thickness, protect against mechanical and bacterial challenges, and enhance aesthetics.
Abutment selection	Choice of material and design for abutments (e.g., custom vs. stock and titanium vs. zirconia).	Influence soft tissue response, improve adaptation, reduce inflammation, and enhance aesthetic outcomes.
Emergence profile	The shape and contour of the implant restoration where it emerges from the soft tissue.	Support natural tissue contouring, distribute forces evenly, prevent recession, and improve aesthetics.
Prosthetic margin placement	Positioning of the prosthetic margin relative to the gingival line (subgingival vs. supragingival).	Avoid subgingival margins to reduce plaque accumulation, facilitate hygiene, and prevent peri-implantitis.
Hygiene protocols	Oral hygiene practices are recommended for patients with dental implants.	Prevent peri-implant diseases, maintain soft tissue health, and ensure long-term success.
Clinical assessments	Regular evaluations of soft tissue health around implants.	Detect and manage complications early, monitor tissue response, and maintain overall implant health.
Gingival margin position	Position of the gingival margin in relation to the implant and restoration.	Affects the visual harmony of the smile, contributes to aesthetic outcomes, and maintains healthy soft tissue levels.
Soft tissue contours	The natural shape and symmetry of soft tissues around implants.	Contribute to pleasing aesthetics, support proper function, and enhance patient satisfaction.
Systemic health	Patient's overall health conditions (e.g., diabetes and smoking).	Influence healing and tissue response, affect integration and stability, and necessitate tailored treatment plans.
Genetic factors	Genetic predisposition affecting soft tissue response and disease susceptibility.	Determine the quality of soft tissue integration, influence healing outcomes, and guide personalized treatment strategies.
Implant diameter and length	Selection based on the clinical situation to ensure adequate support.	Provide structural support, enhance tissue stability, and improve overall implant success.
Placement depth	Vertical positioning of the implant to maintain biological width and tissue coverage.	Prevent soft tissue recession, ensure adequate coverage, and support long-term stability and aesthetics

## Conclusions

Soft tissue concerns are critical to the success of dental implants, affecting both functional and aesthetic outcomes. Understanding cellular and molecular mechanisms, histological traits, and material biocompatibility is critical at the microscopic level. By concentrating on these microscopic factors, dental professionals can improve clinical outcomes and patient satisfaction with dental implant treatments.

Clinical management, prosthetic design, maintenance methods, and aesthetic considerations all play important roles on a macro level. Soft tissue integration at the macroscopic level is critical for dental implants' long-term success. Clinicians can improve soft tissue health and produce optimal aesthetic results by using effective surgical procedures, selecting appropriate prosthetic components, and adhering to rigorous oral hygiene protocols. Understanding the macroscopic elements that influence soft tissue integration allows dentists to provide more thorough care and increase patient satisfaction with dental implants. By addressing these aspects holistically, dental professionals can increase soft tissue integration, patient satisfaction, and long-term success with dental implants. This paper covers soft tissue issues in dental implants from both microscopic and macroscopic viewpoints. Understanding these elements allows dental professionals to better manage soft tissue integration, which improves patient care and treatment outcomes.
